# Supplementary figures and images for: Association network analysis identifies enzymatic components of gut microbiota that significantly differ between colorectal cancer patients and healthy controls
Source: PeerJ. 2019 Jul 29;7:e7315. doi: 10.7717/peerj.7315 (PMC6673421; doi:10.7717/peerj.7315)

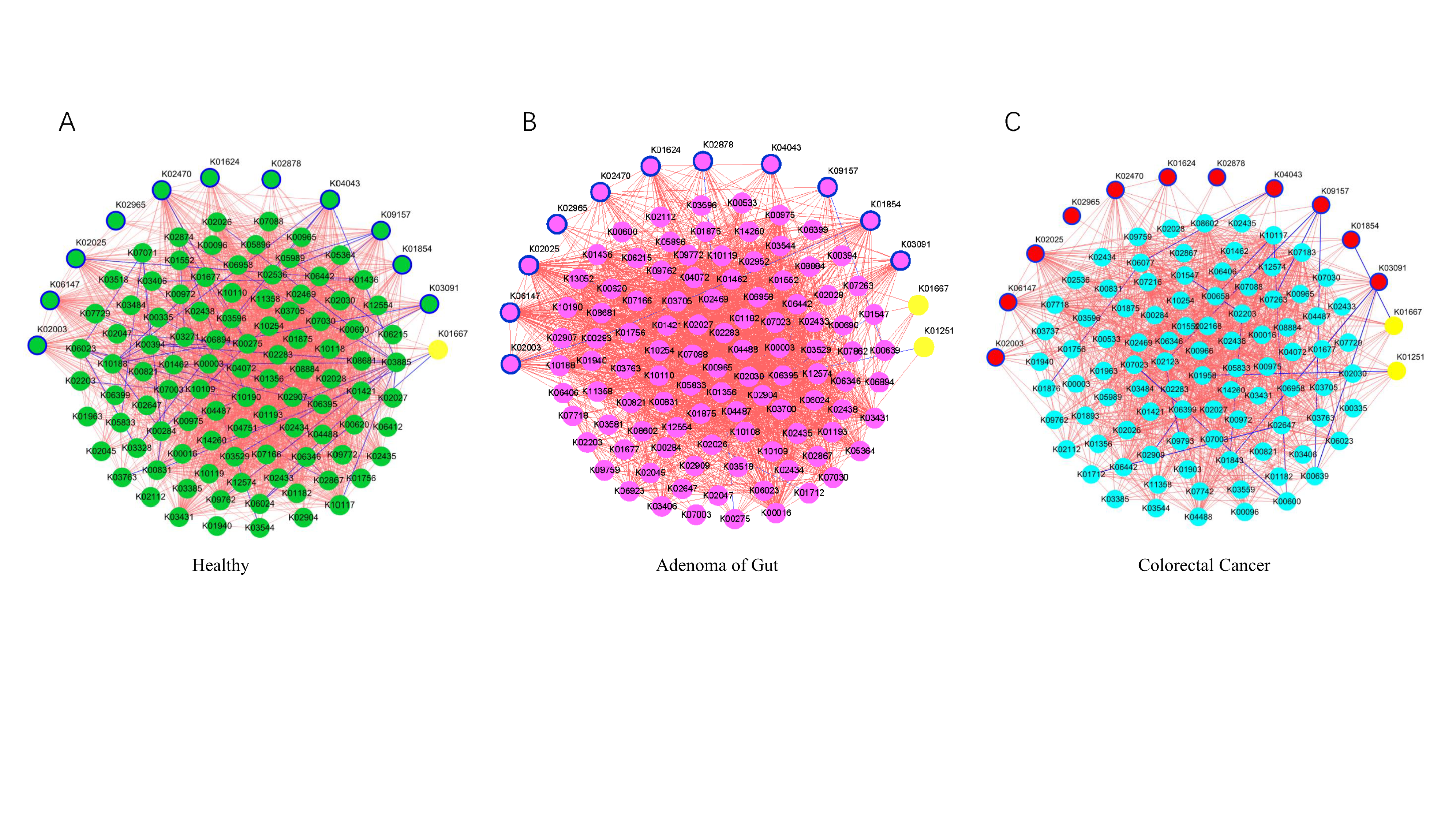

Supplement: Supplemental Information 1 — (A) Metabolic association network of healthy people. (B) Metabolic association network of adenoma patient. (C) Metabolic association network of colorectal cancer patient. In these three networks, the nodes with blue circles represent enzymes enriched in the healthy people, and the nodes with yellow represent enzymes enriched in the colorectal cancer patients, e.g K01251 (adenosylhomocysteinase) was enriched in the adenoma and colorectal cancer samples, but absented in the healthy samples. This enzyme is a biomarker for colorectal cancer. [file peerj-07-7315-s001.png]
